# Retinol-Binding Protein 4 as a Biomarker in Cancer: Insights from a Pan-Cancer Analysis of Expression, Immune Infiltration, and Methylation

**DOI:** 10.3390/genes16020150

**Published:** 2025-01-25

**Authors:** Jia Zhao, Yaxin Liu, Lingqin Zhou, Yi Liu

**Affiliations:** Key Laboratory of Metabolism and Molecular Medicine of the Ministry of Education, Department of Biochemistry and Molecular Biology, School of Basic Medical Sciences, Fudan University, Shanghai 200032, China; jiazhao21@m.fudan.edu.cn (J.Z.);

**Keywords:** RBP4, pan-cancer analysis, prognosis, DNA methylation, biomarker

## Abstract

Background: Retinol-binding protein 4 (RBP4) is primarily recognized for its role in retinoid transport, but has recently been implicated in cancer progression and prognosis. However, a comprehensive pan-cancer analysis of RBP4’s expression, prognostic significance, and functional associations across various cancers is lacking. Methods: We conducted a pan-cancer analysis of RBP4 using data from public databases. RBP4 expression levels were examined in 33 tumor types, and correlations with clinical outcomes, immune cell infiltration, DNA methylation, and gene mutations were assessed. Enrichment analyses of RBP4 and its co-expressed genes were performed to explore associated biological pathways. Additionally, in vitro experiments were conducted to assess the effects of RBP4 on cell migration and proliferation. Results: RBP4 showed differential expression between tumor and normal tissues, with downregulation in 21 cancer types and upregulation in 6. High expression levels of RBP4 were associated with poor overall survival (OS), disease-specific survival (DSS), and progression-free interval (PFI) in specific cancers, notably in BRCA, HNSC, and STAD, whereas it was a favorable prognostic factor in cancers such as KIRP and MESO. RBP4 expression was also associated with immune cell infiltration, particularly with CD4+ Th2 cells and immune checkpoint genes. DNA methylation analysis suggested that the methylation of RBP4 may play a role in its regulatory mechanisms across cancer types. Enrichment analyses revealed that RBP4 and its co-expressed genes are involved in metabolism-related pathways and immune regulation. Functional assays indicated that RBP4 knockdown promoted tumor cell migration and proliferation. Conclusions: This study provides a comprehensive pan-cancer analysis of RBP4, identifying its prognostic potential and possible involvement in tumor immunity and metabolism. Our findings suggest that RBP4 could serve as a novel biomarker and therapeutic target in cancer, although further experimental studies are required to elucidate its precise mechanisms in specific cancer types.

## 1. Introduction

Cancer is a major public health challenge and has become a leading cause of death worldwide. There were approximately 20 million new cancer cases and nearly 9.7 million cancer deaths worldwide in 2022 (both estimates exclude non-melanoma skin cancer) [1]. The development of cancer is driven by multiple factors, with uncontrolled cell proliferation being the primary cause. This uncontrolled growth is primarily attributed to the accumulation of genetic and epigenetic alterations, which together contribute to tumor formation [2]. Given that cancer treatment continues to face significant challenges [3], current therapeutic approaches primarily focus on targeted therapies, with drug development increasingly being aimed at regulating metabolism-related systems [4,5], reducing inflammation [6,7], inhibiting cell proliferation, or blocking protective immune responses [8]. In order to search for new immunotherapeutic targets, more researches are conducted to find key factors influencing tumor progression through immune cell infiltration. In recent years, immunotherapy, especially immune checkpoint blockade therapy has become a major weapon in fighting cancer [9].

Nowadays, with the development and improvement of public databases such as The Cancer Genome Atlas (TCGA), Genotype-Tissue Expression (GTEx), and the Human Protein Atlas (HPA), an increasing number of researchers are using pan-cancer expression analyses to identify new immunotherapy targets and biomarkers [10]. These analyses often focus on evaluating the correlations of gene expression with clinical prognosis and associated signaling pathways.

Retinol-binding protein 4 (RBP4) is the prototype of liposome protein and a 21 kDa protein that is considered a specific carrier of retinol in the blood [11,12]. Defects or mutations in RBP4 can lead to a range of conditions and diseases, primarily due to disrupted retinoid homeostasis. These conditions can affect processes such as embryonic development, vision, metabolism, and cardiovascular health [13]. Moreover, RBP4 has been shown to influence immune and inflammatory mechanisms in adipose and vascular tissues, contributing to the progression of insulin resistance [14,15]. It has also been highlighted in recent studies that RBP4 is of critical importance in cancer cell metabolism. Those fundings suggested RBP4 could be a potential novel biomarker for the diagnosis, treatment, and progression of various cancers, including breast cancer [16], hepatocellular carcinoma [17], and colorectal cancer [18]. The high-fat diet (HFD) promotes colon carcinogenesis, and the key mechanism is that the RBP4–STAR6 pathway serves as a key mechanism for sustaining the self-renewal of colon cancer [19]. In conclusion, increasing evidence suggests that RBP4 might be an emerging biomarker in cancers, and its expression can affect patient prognosis.

As of now, the majority of research has focused on the role of RBP4 in specific types of cancer, with limited exploration of its broader implications. While several pan-cancer analyses of RBP4 have been conducted, such as those utilizing liver tumor cells [17] or focusing on limited cancer types and datasets [17,20], our approach offers significant advantages. We leverage a broader range of cancer types from multiple comprehensive databases, and our study incorporates both in vitro and in silico analyses, providing a more holistic view of RBP4’s role in cancer progression. In this study, we analyzed the mRNA expression levels of RBP4 across 33 cancer types, along with its associations with cancer prognosis, immune-related factors, and DNA methylation. Additionally, we investigated the proteins closely associated with RBP4, constructed protein co-expression networks, and conducted enrichment analyses to identify pathways and biological functions involving these proteins. Moreover, through in vitro experiments, we investigated the function of RBP4 in modulating the development of liver tumor cells.

## 2. Materials and Methods

### 2.1. Data Preprocessing and Analysis of RBP4 Gene in Pan-Cancer

We used the GTEx database to analyze RBP4 expression in normal tissues from healthy individuals, and the expression data of tumor tissues were obtained from the TCGA database. Moreover, we used an online platform UCSC Xena (https://xena.ucsc.edu, accessed on 8 December 2023), which designed for exploring gene expression, clinical, and phenotypic data to obtain the RNA sequencing, somatic mutation, and corresponding clinical data were obtained from TCGA and GTEx databases. Table A illustrates abbreviations for the 33 types of tumors included in this study. The expression level of RBP4 was assessed across 31 normal tissue types and 33 tumor types using the downloaded datasets, with comparative analyses being conducted between cancerous samples, and normal samples were matched across these 33 cancers. After log2-transformation of the expression data, two-tailed *t*-tests were used to perform statistical comparisons of differential expression between tumor and normal tissues. Data analyses were performed in the R software (Version 4.2.3; https://www.R-project.org, accessed on 28 March 2023), and box plots illustrating expression levels were generated using the R package “ggplot2”.

### 2.2. Differences of RBP4 Expression at Protein Level

We obtained immunohistochemistry (IHC) images of RBP4 expression at protein level, including normal and tumor tissues of kidney, liver, colon and stomach from the Human Protein Atlas (HPA, http://www.proteinatlas.org/, accessed on 7 December 2023) for subsequent analysis [21]. All of the antibody catalog numbers of RBP4 were HPA001641.

### 2.3. Analysis of the Relationships Between RBP4, Prognosis, and Clinical Phenotype

After downloaded clinical information from TCGA, survival and clinical phenotype data were screened from each sample. To investigate the potential role of RBP4 expression in cancer prognosis, three indicators were selected, including overall survival (OS), disease-specific survival (DSS), and progression-free interval (PFI). Using the “survminer” package, the best cut-off was calculated based on the median RBP4 expression value, dividing all patient into two groups depending on RBP4 expression level (high or low).

We carried out genetic alterations of RBP4 in pan-cancer by using the cBioPortal database (https://www.cbioportal.org/, accessed on 7 December 2023), a web platform for providing tumors’ genomic information [22]. Based on the data from TCGA PanCancer Atlas Studies, the mutation alterations included mutation, amplification, and deep deletion from 10,967 samples (10,953 patients) were analyzed.

### 2.4. Relationship Between RBP4 Expression and Tumor Immunity

The “xCell” package was used to downloaded the immune-related information and analyze the relationship between RBP4 expression and 37 types of immune cells across 33 cancers. The R packages “ggplot2”, “ggpubr”, and “corrplot” were utilized to assess the correlations between the expression levels of RBP4 and immune cell infiltration. Immune and stromal scores were assessed by xCell algorithm. We also preformed co-expression analysis between RBP4 and 11 immune checkpoint genes [23], and the results were visualized using the R packages “limma”, “corrplot”, and “ggplot2”.

### 2.5. DNA Methylation Analysis of RBP4

A common epigenetic modification, DNA methylation, might act as an early warning marker for a cancer related environment [24]. The UCSC Xena database was searched in an attempt to explore the DNA methylation levels of the RBP4 promoter in specific cancers, aiming to identify the differences between tumor and normal tissues. The R package “biomaRt” was applied to annotate the chip data and to discuss the distribution of methylation probes in chromosomes.

### 2.6. Protein Interaction Network and Enrichment Analysis

A protein interaction network was obtained by using the STRING database (https://string-db.org/, accessed on 13 January 2024), inputting gene names and setting the species to “Human”. We used the default medium confidence score (0.400) in STRING to construct the network. The downloaded protein interaction network was imported into Cytoscape (Version 3.9.1, https://cytoscape.org/, accessed on 13 January 2024) to visualize the PPI network. The Gene Ontology (GO) enrichment analysis was used to represent the various roles of genes in an organism, including the following three: Biological Processes (BPs), Cellular Components (CCs), and Molecular Functions (MFs). Moreover, we applied the Kyoto Encyclopedia of Genes and Genomes (KEGG) enrichment analysis to illustrate the biological pathways in which genes were involved. The “ClusterProfiler” package was used for the GO and KEGG enrichment analyses of the interacting genes obtained from the PPI network. After the GO analysis, five results were plotted for each of the three types of BP, CC, and MF pathways using bubble plots, and the 15 results of the KEGG analysis were plotted using bar charts.

### 2.7. Cell Culture

We obtained Huh7 cell lines from the American Type Culture Collection (ATCC). The fetal bovine serum (10%) and penicillin/streptomycin (1%) were mixed with DMEM complete medium to culture cells. The cells were cultured in a cell incubator set at 37 °C with a 5% carbon dioxide atmosphere. Upon reaching approximately 80% confluence, cells were passaged at a ratio of 1:2 to 1:4 following trypsinization. For transfection, cells were seeded at 50% confluence one day prior and transfected using Lipofectamine 2000. siRNA transfection was conducted at 500 ng siRNA per 10^6^ cells. RBP4 Protein (OkayBio, Nanjing, China, K5916) treatment was applied without transfection reagents at a concentration of 30 mg/mL. As presented in Table 1, the sequences of siRNA are given, offering two different types of siRBP4. 

### 2.8. Cell Wound Healing

To conduct a scratch assay, the back of a six-well plate was marked with evenly spaced straight lines, 1 cm apart, ensuring that each well contained at least five intersecting lines. Cells were seeded uniformly across the six wells. After the cells achieved confluence, a 200 µL sterile pipette tip was used to create a linear scratch in each well, which was then aligned parallel to the horizontal markings. To ensure consistency in the scratch width, the same pipette tip was used for all wells. After scratching, the cells were washed three times with PBS to remove debris, the markings were removed from the underside of the plate, and 2 mL of serum-free medium was added to each well. Images of the scratch areas were captured under a microscope with a magnification of 20× at 0, 24, and 48 h, ensuring that the scratches were centered and vertically aligned. Image analysis was performed to assess cell migration (wound healing rate), which was calculated using the following formula:

cell mobility (wound healing rate) = (initial scratch area − scratch area after × hours)/initial scratch area.

### 2.9. Cell Counting Kit-8 (CCK-8) Assay for Assessing Cellular Proliferation

In a 96-well plate, the cells were seeded at a density of 5000 cells per well, with each well having a volume of 100 μL. At each time point, there were five replicates for each group, and baseline measurements (0 h) were taken after 4 h of incubation. For each measurement, 10 μL of CCK-8 (YEASEN, Shanghai, China, 40203ES60) mixed with 100 μL of serum-free medium was added to each well. After 2 h of incubation, we used a microplate reader to measure the absorbance of the plate, and the wavelength was set at 450 nm. The cells were returned to the incubator, and measurements were repeated at 12 h, 24 h, 48 h, and 72 h to assess the absorbance at multiple time points.

### 2.10. Western Blotting

We used cell lysis buffer to lyse cells for protein extraction. The buffer was composed of 50 mM Tris-HCl (PH 6.8) (Beyotime, Shanghai, China, ST760) and 2% SDS (SINOPHARM, Beijing, China, 30166428) supplemented with 100× PMSF (Amresco, Solon, OH, USA, 329-98-6), Cocktail (YEASEN, Shanghai, China, 20124ES), 50× protease inhibitors (YEASEN, Shanghai, China, 20124ES03), and 50× phosphatase inhibitors (YEASEN, Shanghai, China, 20200ES76). Protein concentrations were determined by making use of a BCA assay kit (Pierce, Rockford, IL, USA, 23225). The whole process was carried out strictly in line with the instructions provided by the manufacturer. Following the BCA assay, proteins were separated using SDS-PAGE and subsequently transferred onto methanol-activated PVDF membranes (Millipore, Billerica, MA, USA). We incubated the membranes with primary antibodies overnight (16 h) at 4 °C, followed by incubation with secondary antibodies (Jackson, Bar Harbor, ME, USA, 115-035-003) at 1:10,000 dilution at room temperature (1h). The primary antibody for RBP4 (Abcam, Cambridge, MA, ab109193) was used at a 1:5000 dilution, and for GAPDH (Proteintech, Rosemont, IL, USA,10494-1-AP), it was used at a 1:8000 dilution. An ECL Western blotting substrate (Share-Bio, Shanghai, China) were used to detect protein bands. 

### 2.11. Statistical Analysis

Image processing and quantitative measurements were performed using ImageJ (Version Java 1.8.0_322, https://imagej.net/ij/, accessed on 8 January 2023) and GraphPad Prism (Version 8.4.2, https://www.graphpad.com/, accessed on 8 January 2023), while statistical analyses and graphical processing were carried out using R. The results were shown as the mean ± standard error of the mean (SEM). Each experimental group underwent analysis through multiple independent replications. For the purpose of evaluating the association between RBP4 expression and clinical characteristic, along with the connection between RBP4 expression and DNA methylation, Spearman’s correlation test was carried out with data from the TCGA and GTEx databases.

Kaplan–Meier survival analyses were conducted to evaluate OS, DSS, and PFI, with log-rank tests being applied to assess statistical significance and the RBP4 expression groups being defined based on the cut-off value. The results were illustrated as survival curves through using the R packages “survival” and “survminer”. Cox proportional hazard regression models were used to calculated hazard ratios (HRs), and these HRs were transformed into their natural logarithms. The regression results were visualized using forest plots generated with the “forestplot” package. In the pathway enrichment analyses, a significance threshold of *p* < 0.05 and a false discovery rate (FDR) of <0.05 were adopted as the criteria. All visualizations, including box plots, scatter plots, and heatmaps, were generated using the R packages “ggplot2”, “survminer”, and “corrplot”.

## 3. Results

### 3.1. Differential mRNA and Protein Expression Levels of RBP4 in Pan-Cancer

**Figure 1:**
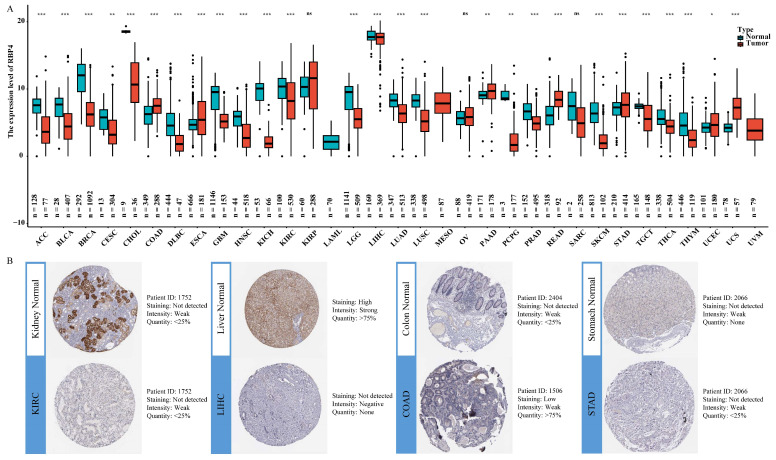
Analysis of RBP4 expression in various types of cancer: (**A**) The differential mRNA expression levels of RBP4 across cancer types. (**B**) The IHC images shows protein expression levels of RBP4in KIRC, LIHC, COAD, and STAD, obtained from the HPA database (*, *p* < 0.05; **, *p* < 0.01; ***, *p* < 0.001; ns indicates no statistical significance).

The results showed that the expression level of RBP4 was upregulated in 6 types of cancer, namely, COAD, ESCA, PAAD, READ, STAD, and UCS, but it was downregulated in 21 other types of cancer compared with normal tissues (Figure 1A). Based on the availability of RBP4-related specimens and the significant differences in IHC staining in the HPA database, we selected two cancer types with elevated RBP4 expression and two with decreased expression for analysis. As shown in Figure 1B, normal liver and kidney tissues had strong RBP4 IHC staining, while tumor tissues had moderate staining. However, in colon and stomach tissue samples, normal tissues exhibited weaker RBP4 staining compared to tumor tissues.

### 3.2. Association Between RBP4 and Clinicopathological Data

**Figure 2:**
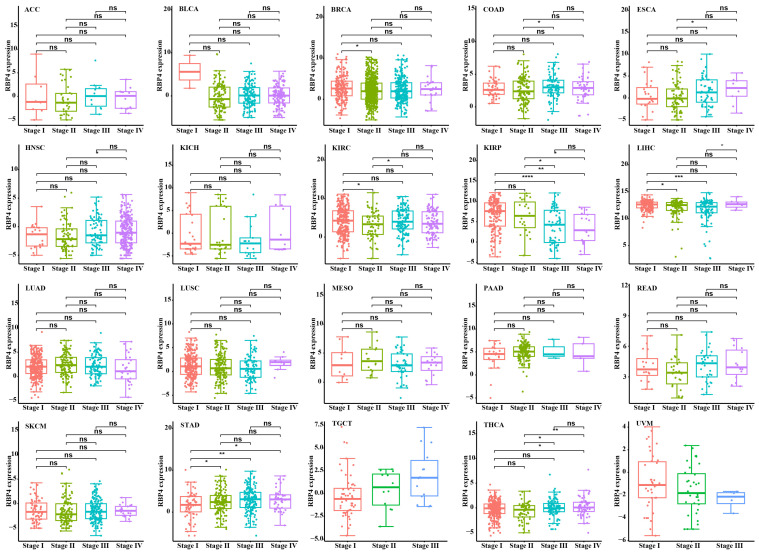
The expression profiles of the RBP4 gene in stages I, II, III, and IV of diverse cancer types derived from the TCGA database (*, *p* < 0.05; **, *p* < 0.01; ***, *p* < 0.001; ****, *p* < 0.0001, ns indicate no statistical significance).

We investigated the relationship between RBP4 and pathological characteristics, and found that the RBP4 expression level was higher in the primary stage of most types of cancer, especially in BRCA, KIRP, and LIHC, but no significant changes were observed in RBP4 expression across the stages in many cancers (Figure 2).

### 3.3. Prognostic Value of RBP4

**Figure 3:**
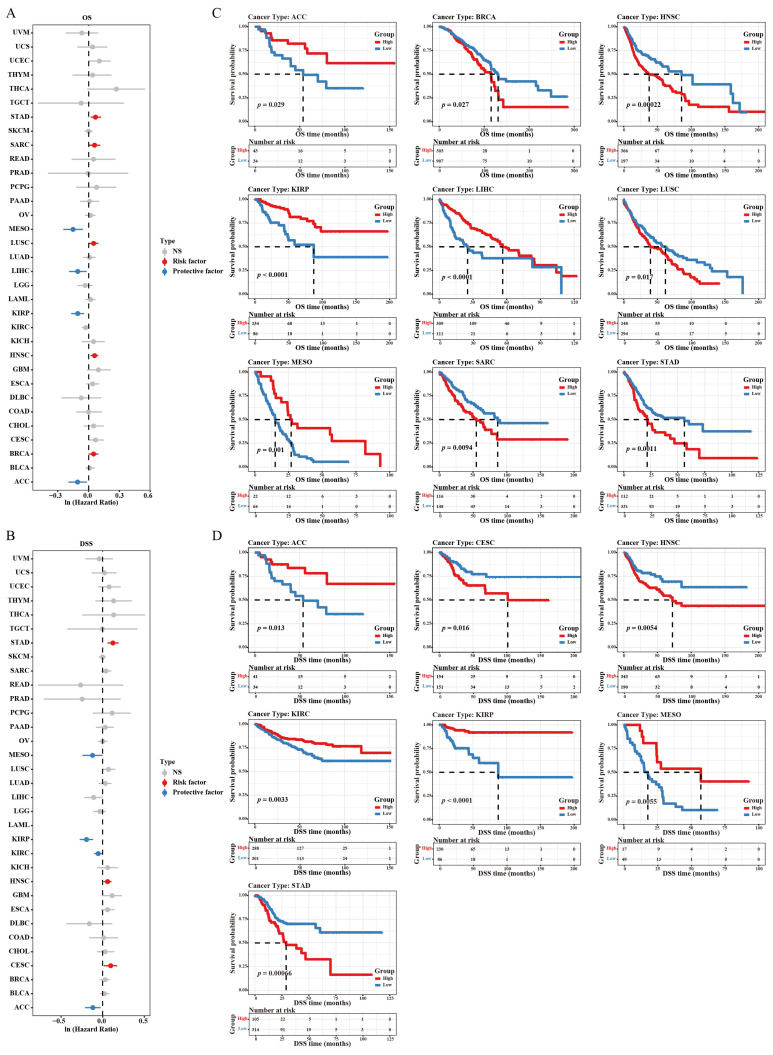
The relationship between RBP4 expression and patient outcomes was analyzed as follows: (**A**) Forest plot illustrating the associations between RBP4 expression and overall survival (OS) across 33 cancer types (risk factor: *p* < 0.05 and Ln HR > 0, protective factor: *p* < 0.05 and Ln HR < 0). (**B**) Kaplan–Meier survival analysis was conducted to illustrate the relationship between RBP4 expression and OS. (**C**) Forest plot displaying the associations between RBP4 expression and disease-specific survival (DSS) across 33 cancer types. (**D**) The association between RBP4 expression and DSS were showed by Kaplan–Meier analysis.

**Figure 4:**
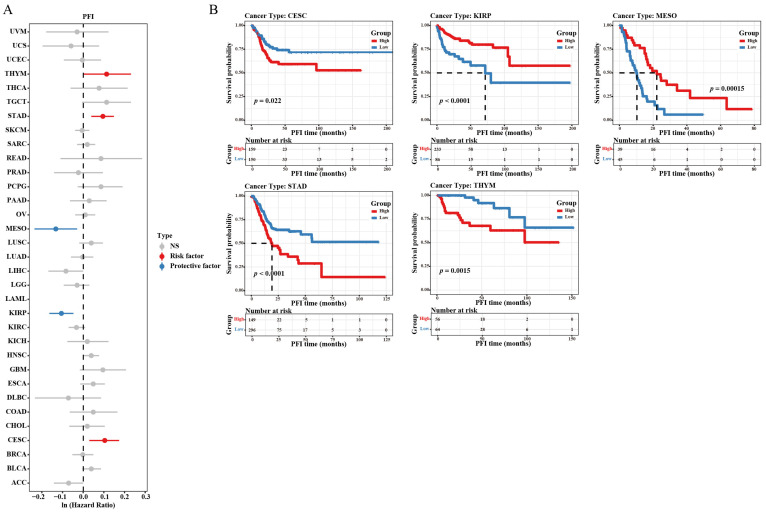
The relationship between RBP4 expression and the progression-free interval (PFI) was analyzed as follows: (**A**) Forest plot illustrating the associations between RBP4 expression and PFI across 33 cancer types (risk factor: *p* < 0.05 and Ln HR > 0, protective factor: *p* < 0.05 and Ln HR < 0). (**B**) Kaplan–Meier survival analysis was conducted to illustrate the association between RBP4 expression and PFI.

The overall survival (OS), disease-specific survival (DSS), and progression-free interval (PFI) were selected to investigate the prognosis of RBP4 in pan-cancer. The analysis of OS showed that high RBP4 expression is a risk factor in BRCA, HNSC, LUSC, SARC, and STAD. However, low expression of RBP4 is a protective factor in ACC, KIRP, LIHC, and MESO (Figure 3A,B). As for DSS, high RBP4 expression had shorter DSS in CESC, HNSC, and STAD, but it had longer DSS in ACC, KIRC, KIRP, and MESO (Figure 3C,D). Moreover, high RBP4 expression was associated with unfavorable PFI in CESC, STAD, and THYM, while low RBP4 expression correlated with favorable PFI in KIRP and MESO (Figure 4A,B). Interestingly, an under expression of RBP4 in KIRP and MESO was linked to longer OS, DSS, and PFI. These findings suggest that RBP4 may serve as a useful prognostic marker for KIRP and MESO.

### 3.4. Correlation Between RBP4 and Tumor Immunity

**Figure 5:**
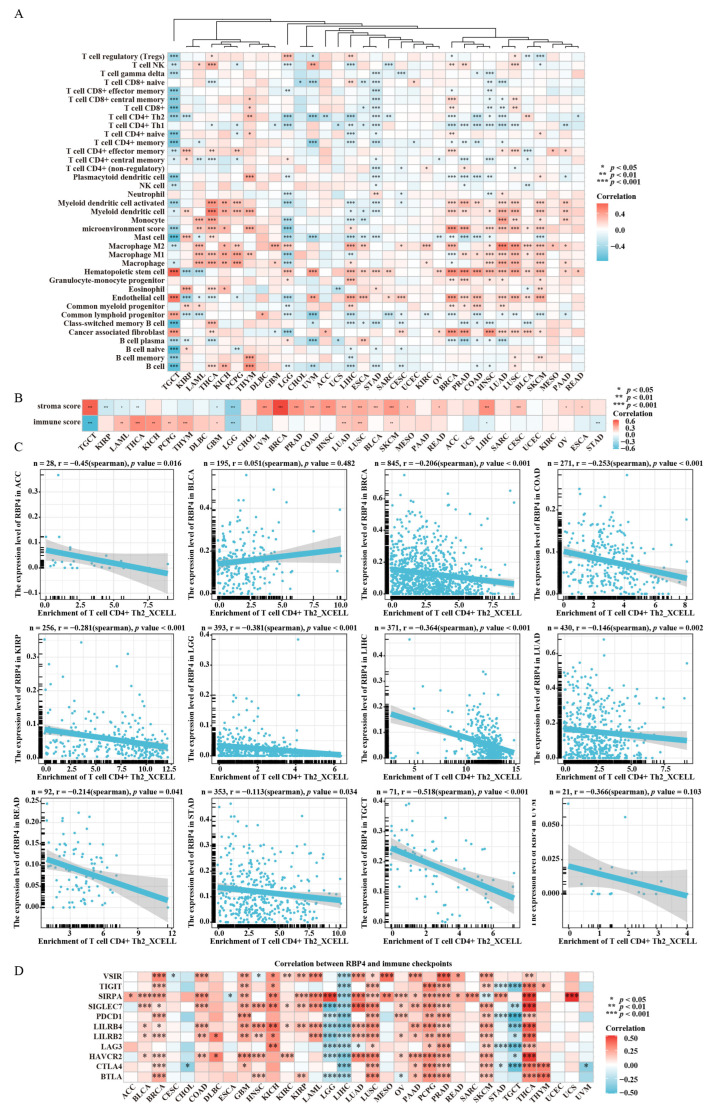
The relationship between RBP4 expression and immune cell infiltration across different cancers was examined as follows: (**A**) Heatmap illustrating the correlation between RBP4 expression and 37 types of immune cells. (**B**) Correlation analysis between RBP4 expression levels and CD4+ Th2 cells in specific cancers. (**C**) Analysis of the relationship between RBP4 expression and immune checkpoint genes. (**D**) The correlation between RBP4 expression and immune checkpoints was showed by heatmap. (*, *p* < 0.05; **, *p* < 0.01; ***, *p* < 0.001).

We investigated that the expression level of RBP4 expression was associated with the infiltration levels of 37 types of immune cells through Spearman correlation analysis. RBP4 exhibited positive or negative correlations with various immune cell types across the majority of cancer types analyzed (Figure 5A). Notably, RBP4 expression showed a strong positive correlation with hematopoietic stem cell infiltration in most cancers. RBP4 was also negatively correlated with CD4+ Th2 T cell infiltration across 33 cancers, with statistically significant correlations being observed in 13 cancer types. Additionally, RBP4 expression levels were positively correlated with the stroma score and immune score in most cancers, indicating their association with the tumor microenvironment (Figure 5B). Taking CD4+ Th2 T cell infiltration as an example, we visualized the significant negative correlations between RBP4 expression and CD4+ Th2 T cells (Figure 5C). Furthermore, RBP4 was positively correlated with immune checkpoints, such as VSIR, TIGIT, SIRPA, SIGLEC7, PDCD1, LILRB4, LILRB2, LAG3, HAVCR2, CTLA4, and BTLA, across many cancers, but it showed negative correlations with immune checkpoints in LIHC and TGCT (Figure 5D).

### 3.5. Mutation Information of RBP4 in Pan-Cancer

**Figure 6:**
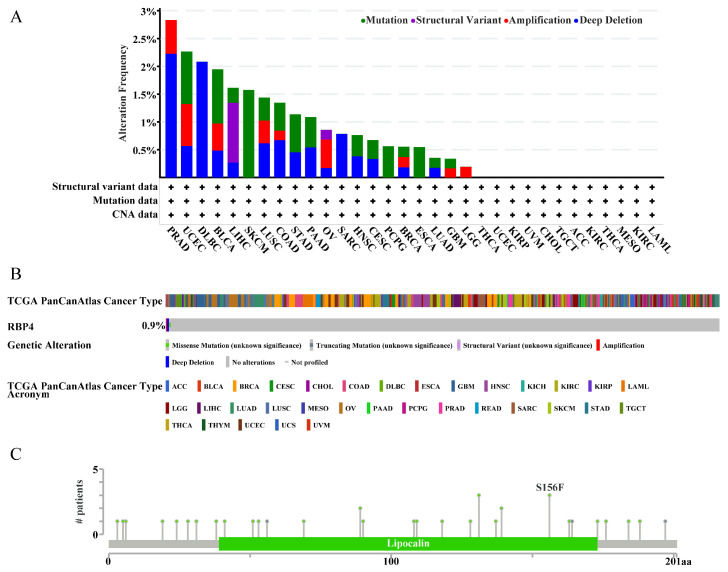
The genetic alterations of RBP4 were illustrated as follows: (**A**) The alteration frequency and mutation types of RBP4 across various cancers, as obtained from cBioPortal. (**B**) A summary of RBP4’s structural variants, mutations, and copy-number alterations in pan-cancer. (**C**) Identification of specific sites associated with different mutation types of RBP4.

We analyzed the alteration status of RBP4 across pan-cancer using data from the cBioPortal database. The most frequent types of RBP4 alterations were amplification, deep deletion, and mutation. As shown in Figure 6A, prostate adenocarcinoma (PRAD) exhibited the highest frequency of RBP4 alterations (2.83%), primarily amplification and deep deletion. The second highest alteration frequency was observed in uterine corpus endometrial carcinoma (UCEC), predominantly involving mutations (0.95%).

According to the database, the presumed copy-number alterations of RBP4 were identified through the Genomic Identification of Significant Targets in Cancer (GISTIC) analysis. The overall mutation frequency for these alterations was 0.9%, and it included various types, such as diploid, gain-of-function, and shallow deletions (Figure 6B). Missense mutations within the lipocalin/cytosolic fatty-acid-binding protein domain were the most common type of RBP4 alteration. Notably, the S156F amino acid substitution was identified as a result of RBP4-related somatic mutations (Figure 6C).

### 3.6. Correlation of RBP4 Expression with DNA Methylation

**Figure 7:**
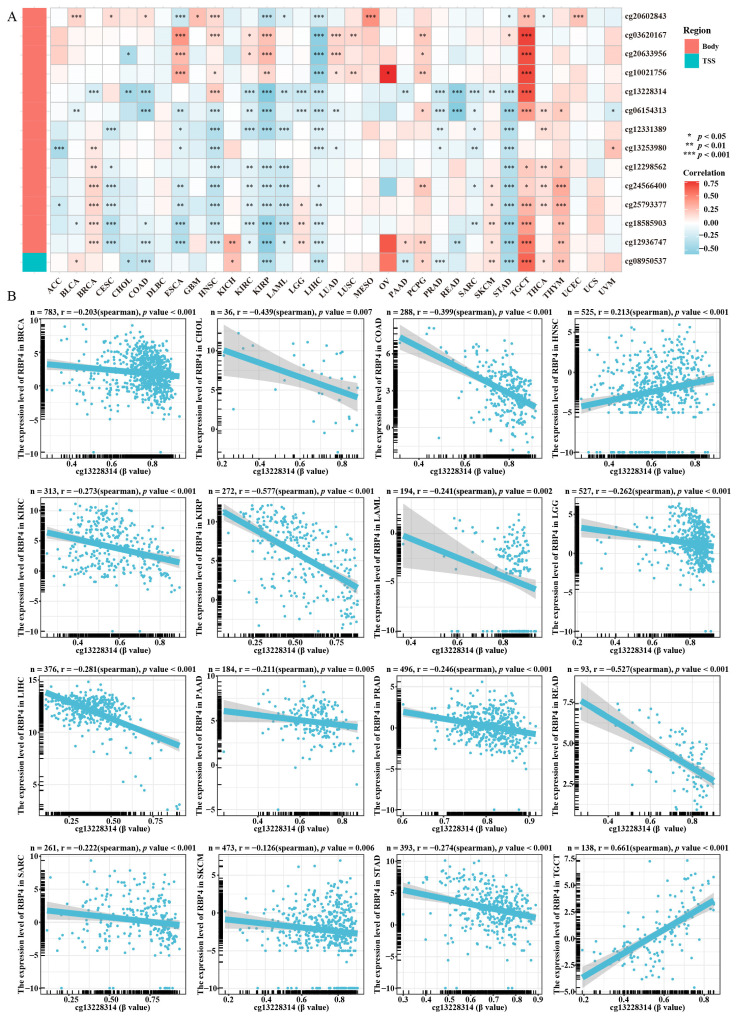
The relationship between RBP4 expression and DNA methylation was examined as follows: (**A**) Analysis of DNA methylation across 33 cancer types. (**B**) Examination of the correlation between RBP4 expression and DNA methylation at the cg13228314 site in BRCA, CHOL, COAD, KIRC, KIRP, LAML, LGG, LIHC, PAAD, PRAD, READ, SARC, SKCM, and STAD.

The R package “biomaRt” was used for DNA methylation level analysis and expression level analysis for each CpG site of RBP4. As shown in Figure 7A, RBP4 has 14 methylation probes, with most methylation regions being located within the gene body, such as cg20602843, cg03620167, cg20633956, cg10021753, cg13228314, cg06154313, cg12331389, cg13253980, cg12298562, cg24566400, cg225793377, cg18585903, and cg12936747; among the methylation regions of the RBP4 gene, only one site, cg08950537, was located in the transcription start site (TSS). Hypermethylated cg13228314 suggested a positive correlation in HNSC and TGCT, but it had a negative correlation in BRCA, CHOL, COAD, KIRC, KIRP, LAML, LGG, LIHC, PAAD, PRAD, READ, SARC, SKCM, and STAD (Figure 7B).

### 3.7. The Enrichment Analysis of Relevant Genes for RBP4

**Figure 8:**
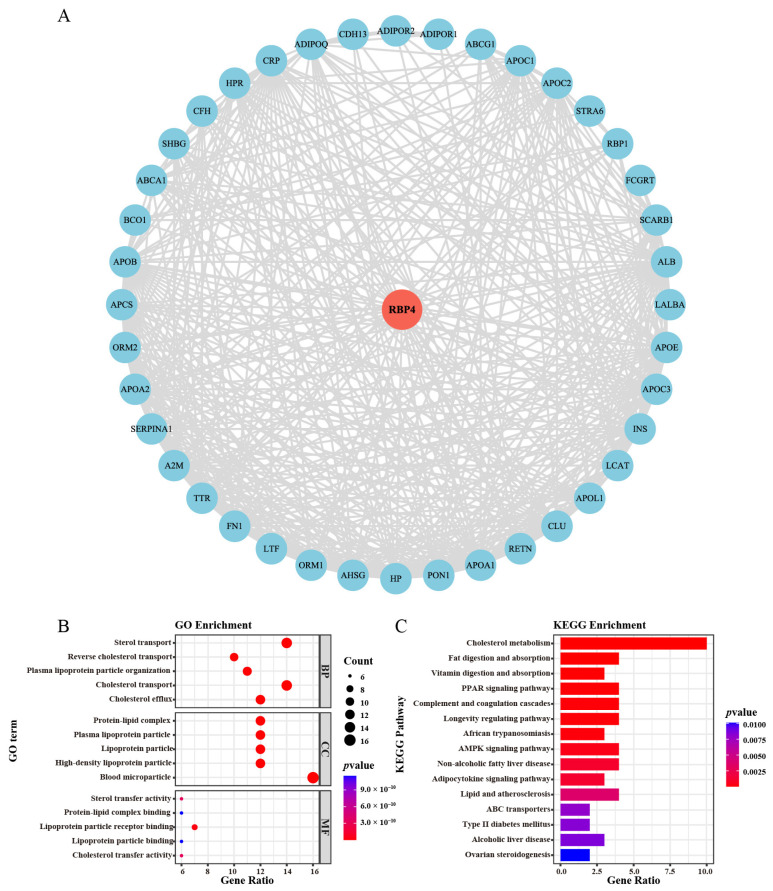
The functional annotation of RBP4 in pan-cancer was conducted as follows: (**A**) PPI network illustrating RBP4 and its forty co-expressed genes. (**B**) GO enrichment analysis of RBP4-related genes, plotting biological processes, cellular components, and molecular functions associated with RBP4. (**C**) KEGG enrichment analysis of RBP4-related genes, identifying key pathways involved in RBP4 function.

By using STRING and Cytoscape, we constructed a protein co-expression network to identify 40 genes co-expressed with RBP4 (Figure 8A).

The GO and KEGG enrichment analyses illustrated the biological functions and pathways associated with RBP4 and its co-expressed genes. The GO analysis showed that these genes are primarily involved in lipid metabolism and transport, including cholesterol and sterol transport, and they are linked to protein–lipid complexes and lipoprotein particles. The molecular functions that were enriched included lipid-binding functions and receptor activity, emphasizing RBP4’s role in metabolic regulation (Figure 8B).

As shown in Figure 8C, the KEGG analysis identified enrichments in 15 pathways, with a particular focus on those related to cholesterol metabolism, fat digestion and absorption, and immune regulation, including the PPAR and AMPK signaling pathways. Additionally, pathways associated with systemic diseases, such as non-alcoholic fatty liver disease, type II diabetes, and atherosclerosis, were also found to be enriched. These results highlight the potential role of RBP4 in lipid metabolism and suggest its broader implications in metabolic disorders and immune regulation.

### 3.8. The Expression of RBP4 Changed the Migration and Proliferation of Cells

**Figure 9:**
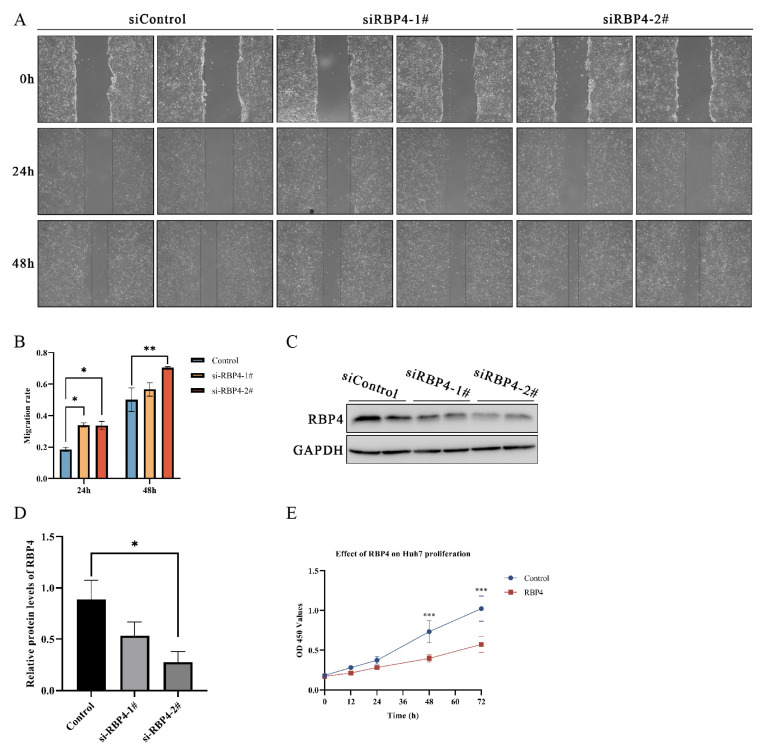
The effect of RBP4 on the migration and proliferation of Huh7 cells: (**A**,**B**) Wound healing assay results indicating that siRBP4 treatment influenced Huh7 cell migration. (**C**,**D**) The expression levels of relevant proteins in Huh7 cells were shown by Western blot analysis. (**E**) Cell proliferation analysis conducted using the CCK-8 assay to evaluate the proliferation capacity of Huh7 cells (*, *p* < 0.05; **, *p* < 0.01; ***, *p* < 0.001).

Given the strong association of RBP4 with metabolic pathways and its significant expression differences in liver cancer, LIHC was selected for further investigation. To investigate whether RBP4 affects the migration ability of liver cancer cells in vitro, we used siRNA to knock down the RBP4 gene in Huh7 cells and conducted a scratch assay to evaluate changes in cell migration. The results indicated that RBP4 knockdown led to an increase in scratch healing and migration rates in both siRNA-treated groups (Figure 9A,B). Notably, both siRBP4-1# and siRBP4-2# effectively knocked down RBP4 expression. Moreover, cells treated with siRBP4-2# showed a statistically significant increase in migration, indicating that RBP4 knockdown enhances tumor cell migration (Figure 9C,D).

To further examine the effect of RBP4 on Huh7 cell proliferation, we used a CCK-8 assay after exogenous RBP4 protein treatment. As shown in Figure 9E, RBP4 protein treatment significantly reduced cell proliferation compared with the control group, with the inhibitory effect becoming more pronounced over time. At 48 and 72 h, the proliferation differences between groups were statistically significant.

## 4. Discussion

RBP4 is primarily synthesized and secreted by the liver and adipose tissue, where it functions as the main carrier of retinol, transporting it from the liver to extrahepatic target cells. This process plays a crucial role in growth regulation and metabolic diseases [25,26,27].

Our work confirmed the significant under expression of RBP4 in 21 cancers and overexpression of RBP4 in six cancers from the TCGA and GTEx databases. Moreover, the IHC images from HPA confirmed this tendency at the protein level in KIRC, LIHC, COAD, and PAAD. The results for liver cancer [17] and pancreatic cancer [28] were similar to those of previous studies; however, Wang et al. demonstrated that RBP4 expression was increased in ovarian cancer [29], which contradicts our present results. This discrepancy may be caused by the fact that the analyzed samples in our study were sourced from large-scale public databases, rather than a limited number of tumor specimens, and the expression of RBP4 in many cancers was reported for the first time. In several tumors, RBP4 expression was lower in the primary stage, but the changes were not statistically significant. The Kaplan-Meier survival analysis was carried out to examine the impact of RBP4 expression on patient survival. The results showed that in STAD, when the expression level of RBP4 was high, it had a positive effect on OS, DSS, and PFI. However, in KIRP and MESO, high RBP4 expression was associated with poor survival outcomes. Moreover, based on univariate and multivariate regression analyses, RBP4 emerged as an independent biomarker in LIHC and PAAD.

Increasing evidence suggests that immune cell infiltration plays a pivotal role in tumor progression and response to immunotherapy [30]. Therefore, understanding the relationship between RBP4 expression and tumor-associated immune cell infiltration is crucial. Our analysis revealed a negative correlation between RBP4 expression and CD4+ Th2 cell infiltration in several cancers from the TCGA database. CD4+ Th2 cells are essential regulators of immunity and inflammation, and their role in shaping the tumor microenvironment has been well documented. Specifically, Th2 and Th17 cells contribute to cancer-associated inflammation, and targeting these cells has been proposed as a strategy for cancer immunotherapy [31]. Notably, Th2 cells are involved in mediating cancer immunity, and blocking TGF-β signaling in CD4+ T cells has been shown to remodel the tumor microenvironment, potentially curbing cancer progression [32]. Moreover, our study underscores the broad relevance of RBP4 in tumor biology. RBP4 expression is closely associated with immune cell activity and immune-related molecules across a wide range of cancers. The co-expression of RBP4 with checkpoint receptor proteins was also revealed in our study. These results illustrate that the expression of RBP4 could be associated with immune infiltration of the tumor cells and influence patient prognosis. Additionally, these findings indicated RBP4 might have the potential to be a new target for the immunosuppressants.

DNA methylation plays a pivotal role in cancer, often silencing genes that are typically active in healthy tissues, particularly tumor suppressor genes [33]. We observed that the RBP4 promoter methylation level was significantly and negatively related to most tumor tissues but was positively related to TGCT. While promoter demethylation is generally associated with gene activation, the downregulation of RBP4 in many cancers, despite the generally demethylated promoter, suggests that additional regulatory mechanisms may be involved. These findings raise the possibility that other epigenetic factors or post-transcriptional regulation may play a role in modulating RBP4 expression in cancer.

In our study, the results of the GO and KEGG analyses showed that RBP4-related genes are predominantly involved in lipid metabolism, transport processes, immune regulation, and cell death pathways, such as the PPAR signaling pathway and the longevity regulation pathway. Previous studies have confirmed that RBP4 is primarily associated with biological processes related to lipid metabolism [34,35], supporting that RBP4 may influence tumor biology through metabolic reprogramming. These findings imply that RBP4 plays a crucial role in regulating both the metabolic environment and immune response in tumors. Further investigations are required to explore how targeting RBP4 can be therapeutically beneficial in oncology.

In our in vitro studies, we observed that RBP4 regulates liver cancer cell migration and proliferation. These findings are in line with other studies that suggest that RBP4 plays a role in tumor metastasis and cell growth in various cancer types. Additionally, studies have shown that certain retinoid drugs can inhibit tumor cell proliferation [36], further suggesting that RBP4 may act as a modulator of both metastatic potential and cell proliferation, particularly in the context of liver cancer. All in all, these results underscore how crucial RBP4 is in the development of liver cancer and point out its promise as a viable therapeutic target.

## 5. Conclusions

In conclusion, our pan-cancer analysis of RBP4 demonstrates its differential expression between tumor and normal tissues, highlighting associations with clinical prognosis and DNA methylation patterns. These findings suggest that RBP4 may serve as an independent prognostic factor across various cancer types, with expression levels potentially influencing prognostic outcomes, warranting further research on its specific roles in individual cancers. The enrichment analysis suggests that RBP4 may influence tumor cell biology through metabolism-related mechanisms. Although our study provides insights into the potential impact of RBP4 on tumor cell migration and proliferation, further experimental validation is essential to elucidate the underlying molecular mechanisms.

## Figures and Tables

**Table 1:** The sequences for the siRNAs in this study.

	Forward Primer	Reverse Primer
siRBP4-1#	5′-CGAAGGCTCAGTTTCTCTT-3′	5′-AAGAGAAACTGAGCCTTCG-3′
siRBP4-2#	5′-TCCTGGGACGGTTCAAGTTCTACTT-3′	5′-AAGTAGAACTTGAACCGTCCCTGGA-3′

## Data Availability

All relevant data are within the manuscript.

## Abbreviations

**Table A.** The abbreviations and corresponding full names of the 33 cancers [37].

**Abbreviation**	**Cancer Type**
ACC	Adrenocortical carcinoma
BLCA	Bladder urothelial carcinoma
BRCA	Breast invasive carcinoma
CESC	Cervical squamous cell carcinoma and endocervical adeno carcinoma
CHOL	Cholangio carcinoma
COAD	Colon adenocarcinoma
DLBC	Lymphoid neoplasm diffuse large B-cell lymphoma
ESCA	Esophageal carcinoma
GBM	Glioblastoma multiforme
HNSC	Head and neck squamous cell carcinoma
KICH	Kidney chromophobe
KIRC	Kidney renal clear cell carcinoma
KIRP	Kidney renal papillary cell carcinoma
LAML	Acute myeloid leukemia
LGG	Brain lower-grade glioma
LIHC	Liver hepatocellular carcinoma
LUAD	Lung adenocarcinoma
LUSC	Lung squamous cell carcinoma
MESO	Mesothelioma
OV	Ovarian serous cystadenocarcinoma
PAAD	Pancreatic adenocarcinoma
PCPG	Pheochromocytoma and paraganglioma
PRAD	Prostate adenocarcinoma
READ	Rectum adenocarcinoma
SARC	Sarcoma
SKCM	Skin cutaneous melanoma
STAD	Stomach adenocarcinoma
TGCT	Testicular germ cell tumors
THCA	Thyroid carcinoma
THYM	Thymoma
UCEC	Uterine corpus endometrial carcinoma
UCS	Uterine carcinosarcoma
UVM	Uveal melanoma
